# Mesopore-dominant nitrogen-doped carbon with a large defect degree and high conductivity *via* inherent hydroxyapatite-induced self-activation for lithium-ion batteries†

**DOI:** 10.1039/c8ra02034e

**Published:** 2018-03-28

**Authors:** Hanwei Wang, Chengmin Sheng, Tailong Cai, Chunde Jin, Qingfeng Sun, Chao Wang

**Affiliations:** School of Engineering, Zhejiang A&F University Hangzhou 311300 China qfsun@zafu.edu.cn chaowangzafu@163.com

## Abstract

In this study, N-doped mesopore-dominant carbon (NMC) materials were prepared using bio-waste tortoise shells as a carbon source *via* a one-step self-activation process. With intrinsic hydroxyapatites (HAPs) as natural templates to fulfill the synchronous carbonization and activation of the precursor, this highly efficient and time-saving method provides N-doped carbon materials that represent a large mesopore volume proportion of 74.59%, a high conductivity of 4382 m S^−1^, as well as larger defects, as demonstrated by Raman and XRD studies. These features make the NMC exhibit a high reversible lithium-storage capacity of 970 mA h g^−1^ at 0.1 A g^−1^, a strong rate capability of 818 mA h g^−1^ at 2 A g^−1^, and a good capacity of 831 mA h g^−1^ after 500 cycles at 1 A g^−1^. This study provides a highly efficient and feasible method to prepare renewable biomass-derived carbons as advanced electrode materials for the application of energy storage.

## Abstract 2

A hydroxyapatite-induced self-activation method has been used to prepare nitrogen-doped mesopore-dominant carbon. The carbon has abundant macro/mesopores, high conductivity, and favorable defects and exhibited high-performance in LIBs.

## Graphical abstract

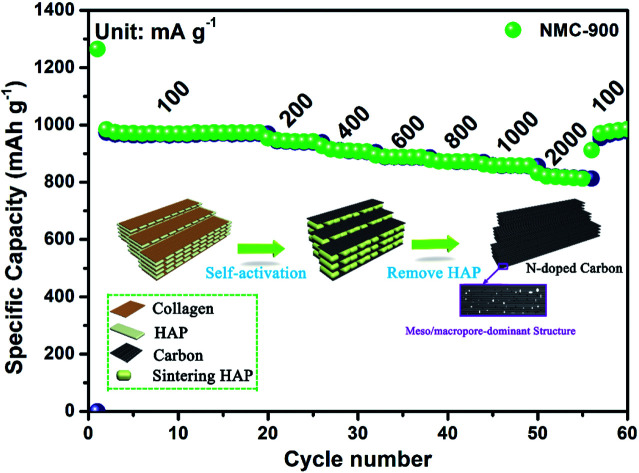

## Introduction

The high demand for high-performance and low-cost lithium-ion batteries (LIBs) has become one of the greatest societal issues because of the boom in electric-equipment in recent decades. Due to their wide availability, economy, desirable safety, and electrochemical stability, carbon-based materials have attracted extensive interest as electrodes in LIBs.^1,2^ However, both low Li^+^ diffusion kinetics and a theoretical capacity of 372 mA h g^−1^ of the commercial graphitic electrodes limit their application in advanced electric-equipment.^3^

Currently, biomass-derived activated carbon with favorable molecular microstructures and architectures, especially hard carbon,^4^ is attracting significant attention as a promising anode in LIBs because of its low price, rich source, abundant defects, and high Li storage capacity.^5^ However, unmanageable porosity and poor conductivity still limit its further application in high-performance LIBs. The conventional production of activated carbon includes two major methods: chemical/physical activation and direct carbonization for biomass materials.^6^ The chemical/physical activators, *e.g.* KOH,^7^ ZnCl_2_,^8^ H_3_PO_4_,^9^ and CO_2_,^10^ are applied to erode the structure of the carbon materials. However, the excessively high proportion of micropores that appears in the produced carbons is usually unfavorable for obtaining a reversible high capacity in the LIBs. In a direct carbonization method, although the obtained carbons generally do not have well-developed porosity, some biomass-derived carbons still present favorable porosity with a relatively complete architecture.^6,11,12^ It is generally believed that intrinsic activators, such as calcium carbonate, hydroxyapatite, group I and II elements, and so on, within the biomass (*e.g.* seaweeds and bones) contribute to form these unique features.^13–15^ Importantly, the pore structure within carbon is usually restricted from the intrinsic activators. This means that selecting a kind of biomass as a precursor with an appropriate intrinsic activator is an easy and effective way to prepare high performance carbon with favorable porosity and a complete architecture.

Tortoises (*Chinemys reevesii*) have had thousands of years of history in China as a source of human food. However, almost half the weight of the shells within the whole tortoise is generally discarded as garbage. For animal bones, collagen and hydroxyapatite (HAP, Ca_5_(PO_4_)_3_(OH)) are two major components of the bone and act as a carbon source and an activating agent, respectively. The plate-shaped HAP within the bones as templates can contribute an abundant mesoporous structure to the final activated carbon. HAP as a more moderate activator helps carbon maintain a relatively complete architecture favorable for achieving high conductivity. Herein, a simple and self-activation method through direct carbonization of the tortoise shells was employed to prepare nitrogen-doped mesoporous carbons (NMCs). The self-activation mechanism of the bone was explored during the carbonization process (600 °C–1000 °C). The as-prepared NMCs possess a high ratio of mesoporous volume (74.59%), an excellent conductivity of 4382 S m^−1^, and a good nitrogen content of 3.84 at% and exhibit high initial coulombic efficiency, remarkable rate capability, and long cycling stability in LIBs. This study provides a feasible method using HAP as templates to prepare a mesoporous-dominant carbon that has superior application potential as a high-rate performance electrode material in the energy-storage field.

## Experimental

### Chemical reagents

The discarded tortoise shells (*Chinemys reevesii*) were obtained from the waste of a packing house in Shangdong, China. Hydrochloric acid (HCl, 37 wt%), polyvinylidene fluoride (PVDF), super-P carbon black, and *N*-methyl-2-pyrrolidone (NMP) were purchased from Aladdin Chemistry Co., Ltd.

### Preparation of NMCs

The tortoise shells were heated at 600, 700, 800, 900, and 1000 °C under a N_2_ atmosphere for 2 h (at the heating rate of 4 °C min^−1^). The obtained products were completely washed 5 times with a 1 M HCl solution and deionized water. The as-prepared carbon materials were dried at 120 °C for 24 h and ground to a powder in the mortar. These carbon materials were denoted as NMC-600, NMC-700, NMC-800, NMC-900, and NMC-1000.

### Characterization

The structures of the samples were analyzed by X-ray diffraction (XRD, Rigaku, D/MAX 2200) at the scan rate (2*θ*) of 4° min^−1^ ranging from 10° to 80°, operating with Cu Kα radiation (*λ* = 1.5418 Å) with an applied current of 30 mA and an accelerating voltage of 40 kV. The morphologies of the samples were observed by scanning electron microscopy (SEM, FEI Quanta FEG 250) and transmission electron microscopy images (TEM, FEI Tecnai G2 F20). The porosities of the samples were studied by nitrogen adsorption–desorption isotherms obtained *via* a Micromeritics Tristar 3000 system using vacuum-degassed samples (300 °C for at least 4 h) at 77.4 K. X-ray photoelectron spectrometer analysis (XPS, ESCALAB 250 XI) was used to determine the elemental composition and functional groups of the samples. Raman spectroscopy (Renishaw 1000NR) was performed at an excitation wavelength of 633 nm at room temperature. Thermogravimetric (TG, TG209F1 Netzsch) analysis was performed at a flow rate of 40 mL min^−1^ under a N_2_ atmosphere. Fourier transform infrared spectroscopy (FTIR, Magna-IR 560, Nicolet) was used to analyze the functional groups of the samples.

### Electrochemical measurements

The slurry of the electrode material (weight active material : weight super-P : weight PVDF = 80 : 10 : 10) was pasted on a Cu foil (*R* = 6 mm) and then dried at 100 °C for 48 h in a vacuum oven. The weight of the active material was 1.6–2 mg cm^−1^ on a single electrode. A pure lithium metal foil as the counter electrode, polypropylene (PP) microfiber filter film (Celgard 2500, 25 μm) as the separator, and 1 M LiPF_6_ (ethylene carbonate : dimethyl carbonate : diethyl carbonate = 1 : 1 : 1 by weight) as the electrolyte were assembled into cells (CR2016 type cells). The charge/discharge experiments of the cells were conducted using a Land Battery Test System (LAND CT2001A) in the potential range of 3.0–0.01 V. CV experiments were performed using a CHI66E electrochemical workstation at the scan rates of 0.1–1 mV s^−1^.

## Results and discussion

**Fig. 1:**
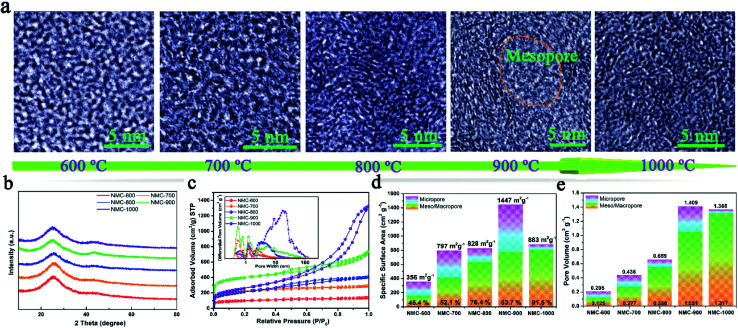
(a) The changing process of the NMCs illustrated by HRTEM images. (b) XRD patterns of the NMCs. (c) Nitrogen sorption isotherms of the NMCs. Inset: pore-size distribution curves of the NMCs. (d) SSA and (e) pore volume of the NMCs.

Tortoise shells were directly carbonized at different temperatures ranging from 600 °C to 1000 °C. The corresponding products were named as TC-600, TC-700, TC-800, TC-900, and TC-1000. After removing HAP by HCl (1 M), the NMC materials were prepared. The SEM image of NMC-900 as a representative is displayed in Fig. S1,† revealing numerous nano-size pores formed upon removing HAP. The TEM images in Fig. S2a and b† showed the inferior mesoporous property of the NMC-600 and NMC-700. Then, plentiful round holes (width < 100 nm) appeared in the NMC structure at higher temperatures, which were formed from regrowth and aggregation of HAP. The HRTEM images of both NMC-600 and NMC-700 in Fig. 1a showed no obvious graphitic microstructures.^16^ With an increase in temperature to 800 °C, parallel carbon layers without a very long-range structure were generated on the carbon surface (NMC-800) due to the reduced heteroatoms and rearranged C atoms. When the temperature was 900 °C, the elliptic mesoporous formed by encircling of relative ordered carbon layers appeared in the NMC-900 sample, which were able to offer large electrode/electrolyte interfaces. At 1000 °C, disordered and compact turbostratic graphitic microstructures with very short-range carbon layers were observed in the sample, where there were no obvious micropores. All the XRD patterns of the NMCs in Fig. 1b displayed an obvious graphitic stacking peak (002) and graphitized peak (101) at 25.0° and 43.4°, respectively, showing the typical hard carbon structure of the NMCs.^23^ The broad (002) diffraction peaks in the patterns implied the presence of graphitic edges within the carbon, which were beneficial for the insertion/extraction of Li^+^, promoting the Li^+^ storage property.^17^

The N_2_ sorption curves of the NMCs are displayed in Fig. 1c, showing the typical type II isotherms of the NMC-600 and NMC-700 as well as type IV isotherms of the other samples. The high adsorption of the NMC-900 at low pressures (*P*/*P*_0_ < 0.1) indicated the presence of abundant micropores. Interestingly, the shape of the type-H4 hysteresis loop in the NMC isotherms gradually enlarged as the temperature increased; this indicated the enlarged size of the mesoporous in the samples.^7^ The pore-size distributions of the NMCs shown in the inset of Fig. 1c maintained a high consistency with these results. In the microporous region (<2 nm), the NMC-900 exhibited highest differential pore volume ascribed to the activation of CO_2_ (C + CO_2_ → 2CO).^18^ In the mesoporous region (2 nm < size < 100 nm), the average pore size of the NMCs gradually diffused to a higher pore width as the temperature increased; this could be attributed to the growth of HAP. The SSA of the NMCs with the percentage of the mesoporous is described in Fig. 1d, showing a high percentage of the mesoporous ranging from 45.4% to 91.5% within the whole pore. The SSA values of the NMC-600, NMC-700, NMC-800, NMC-900, and NMC-1000 were 356, 797, 828, 1447, and 883 m^2^ g^−1^, respectively. The SSA of the mesoporous increased as the temperature increased; this indicated that the growth of HAP at high temperatures not only enlarged the pore sizes, but also enhanced the SSA of the mesoporous. The mesopores within carbon could effectively store electrolyte as reservoirs, which were beneficial to reduce the ion-diffusion resistance and provide rapid Li^+^ transfer pathways.^7^ Although the NMC-1000 had a higher mesoporous surface area, a very low microporous surface area of 85 m^2^ g^−1^ limited the diffusion of Li^+^ and electrolyte within carbon.^2^ As shown in Fig. 1e, the upward tendency of the mesoporous volume values of the NMCs was consistent with the mesoporous surface area. Notably, the maximum pore volume of 1.409 with 74.59% of the mesopores of the NMC-900 indicated a mesoporous-dominant structure.

**Fig. 2:**
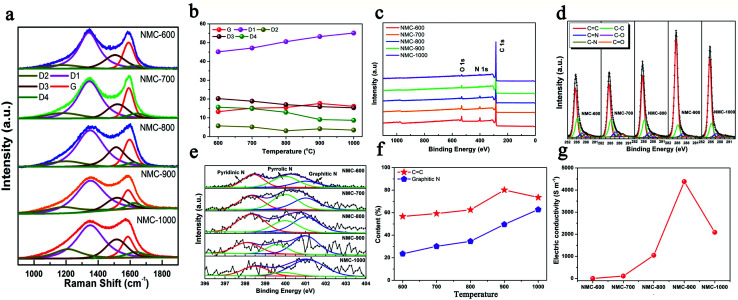
(a) Raman spectra and (b) the contents of Gaussian peaks of NMCs. (c) Survey XPS spectra, (d) high-resolution C 1s spectra, and (e) high-resolution N 1s spectra of the NMCs. (f) The contents of C=C and graphitic N of the NMCs. (g) Electrical conductivities of the NMCs.

Moreover, five peaks were obtained by fitting the Raman spectra of the NMCs in Fig. 2a as follows:^19^ (1) G band at ∼1590 cm^−1^ (ideal graphitic lattice); (2) D1 band at ∼1350 cm^−1^ (graphene layer edges); (3) D2 band at ∼1640 cm^−1^ (surface graphene layers); (4) D3 band at ∼1510 cm^−1^ (amorphous carbon); and (5) D4 band at ∼1200 cm^−1^ (heteroatom contents and polyenes). Fig. 2b shows the contents of the five peaks in the Raman spectra. The growth of the D1 content was faster than that of the G from 600 to 1000 °C; this indicated the formation of more defects. These defects within carbon played key roles in the storage and diffusion of Li^+^ ions.^5^ The decreased G contents of NMC-1000 could be imputed to the overgrowth of HAP that led to the collapse of the microporous structure. Moreover, the gradual reduction in the content of the D4 was caused by the decrease in the contents of N and O elements. As shown in Fig. 2c, the diminishing N 1s (532 eV) and O 1s (400 eV) peaks of the XPS spectra with the increasing temperature also revealed the decreased contents of the N and O elements. The highly carbonized NMC materials at 900 and 1000 °C showed two high C element contents of 92.45% and 93.63% (Table S1†), respectively, which were one of the foundations for the high electrical conductivity of the carbon materials. The corresponding high-resolution C 1s spectra of the NMCs in Fig. 2d could be fitted by six characteristic peaks centered at approximately 284.6, 285.2, 286.0, 286.7, 287.9, and 289.2 eV, corresponding to the C=C, C–C, C=N, C–O, C–N, and C=O bonds, respectively.^20^ The high-intensity and sharp C peak of the NMC-900 implied a high content of the C=C band within the sample. Fig. 2e shows that all the N 1s spectra of the NMCs are composed of pyridinic N, pyrrolic N, and graphitic N at approximately 398.4, 400.0, and 401.0 eV, respectively.^21^ The corresponding contents of the different N s are displayed in Fig. S3.† Among them, pyridinic N showed a high content ranging from 36.6 to 27.2% when the temperature increased, which had a heavy influence on the lithium storage properties. Additionally, it was generally speculated that the graphitic N and C=C band played two critical roles in the electrical conductivity of carbon (Fig. 2f). NMC-900 had a higher C=C band content of 80.13% as well as a superior graphitic N content of 49.60%. The decrease in the C=C band content of NMC-1000 might be caused by the destruction of structure due to the overgrowth and aggregation of HAP. The features of superior C=C band and graphitic N content, a very low O content, and a relatively ordered structure made the NMC-900 possess a superior conductivity of 4382 S m^−1^ (Fig. 2g and Table S1†). Thus, NMC-900 might be a promising electrode material in LIBs because of its superior conductivity, favorable structured defects, mesoporous-dominant porosity, and reasonable pore structure.

**Fig. 3:**
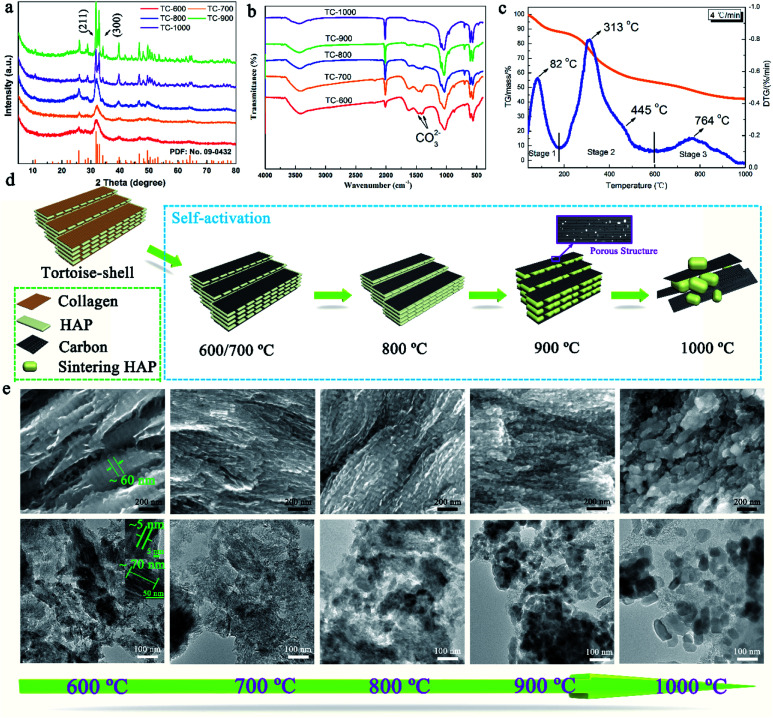
(a) XRD patterns of the TC at different temperatures (600–1000 °C). (b) FTIR spectra of the TC at different temperatures (600–1000 °C). (c) TG/DTG curves of the tortoise shell at the heating rate of 2°C min^−1^ under a N_2_ atmosphere. (d) The self-activation mechanism of the tortoise shells. (e) The changing process of the TC illustrated by SEM and corresponding TEM images.

Then, TC-*X* (*X* = 600, 700, 800, 900, and 900) was further studied for understanding the formation mechanism of NMC. The XRD patterns of the TC and the tortoise shells are presented in Fig. 3a and S4,† showing a typical hydroxyapatite (Ca_5_(PO_4_)_3_(OH)) structure (PDF: no. 09-0432).^22,23^ Broad and weak peaks of HAP were observed for the tortoise shells at 600–800 °C because of their poor crystallinity and/or the small scale of crystals for HAP. When the temperature increased, the (211) (2*θ* = 31.9°) and (112) (2*θ* = 23.3°) reflections became clearer and sharper. According to the XRD patterns, the calculated crystallite sizes of the NMC-800, NMC-900, and NMC-1000 were 8.0, 26.7, and 109.4 nm, respectively, which indicated the remarkable enhancement of crystallization degree as well as the regrowth of the HAP crystals.^24^ Interestingly, the carbonate groups at 1471 cm^−1^ and 1398 cm^−1^ were only observed in the FTIR patterns of TC-600, TC-700, and TC-800 (Fig. 3b).^25^ The corresponding Ca/P ratio of 2.01, 2.08, and 2.04 was obtained from the ICP-MS tests. These results indicated the presence of carbonate within the TC-600, TC-700, and TC-800 and also implied the decomposition of carbonate at higher temperatures.^26^ The carbonate peaks did not appear in the XRD patterns; this were possibly obscured because of the presence of HAP, poor crystallinity, and small content. As shown in Fig. 3c, the thermogravimetric (TG) and differential thermal gravimetric (DTG) curves of the tortoise shells could be divided into three steps. The first stage went from 30 °C to 175 °C. A small weight loss of 11.94% and a strong peak at 82 °C were observed in TG and the corresponding DTG curves, which could be ascribed to the evaporation of the surface adsorbed water and slight volatiles.^26,27^ The second stage ranging from 175 °C to 600 °C indicated the major pyrolysis and carbonization process of collagen with a high mass loss of 34.54%; thus, a maximum peak at 313 °C and a shoulder peak at 445 °C appeared in the DTG curve. The last stage after 600 °C showed a low mass loss of 11.13% and a broad peak at 764 °C in the DTG curve. Specially, the main mass loss of 7.86% occurred at 700–900 °C because of the decomposition of carbonate and further pyrolysis of the organic substance-derived carbon, which could cause changes in the structure and porosity of carbon.^26^

The self-activation mechanism images of the tortoise shells are illustrated in Fig. 3d. The SEM and corresponding TEM images of the TC materials in Fig. 3e show a unique structure and have been employed to study the self-activation mechanism of the tortoise shells. At the temperatures of 600 and 700 °C, the TC showed a regular structure that consisted of paralleled and continuous plates, as shown in the SEM image. The corresponding TEM image revealed that the plate-shaped HAP with an approximate size of 70 × 60 × 3 nm was surrounded by carbon. When the carbonization temperature was increased to 800 °C, the TC showed a similar structure in the SEM image. However, the regular plate-shaped HAP was not clearly observed in the TEM image; this implied the structure change of HAP. As the temperature increased to 900 °C, the continuous plate-shaped HAP was completely replaced by diminutive nanoparticles with a relatively ordered structure. The nanoparticles resulted from the morphological change of HAP during the regrowth process.^27^ It is worth noting that the carbon surrounding the HAP nanoparticles in the TEM image displays a rich mesoporous structure favorable for the fast transport of Li ions. This might be caused by the activation of CO_2_ from the decomposition of carbonate as well as the variation in the HAP structure due to crystal regrowth.^23^ At 1000 °C, further growth and aggregation of the HAP crystals caused the TC-1000 to show a relatively disordered structure, and the surface was densely and randomly covered by large HAP particles, as shown in the SEM image. The TEM image of the TC-1000 proved the further growth of the HAP nanoparticles. The changes in HAP with the increasing temperature, as shown in Fig. 3e, were highly consistent with the results of the calculated crystallite size.

**Fig. 4:**
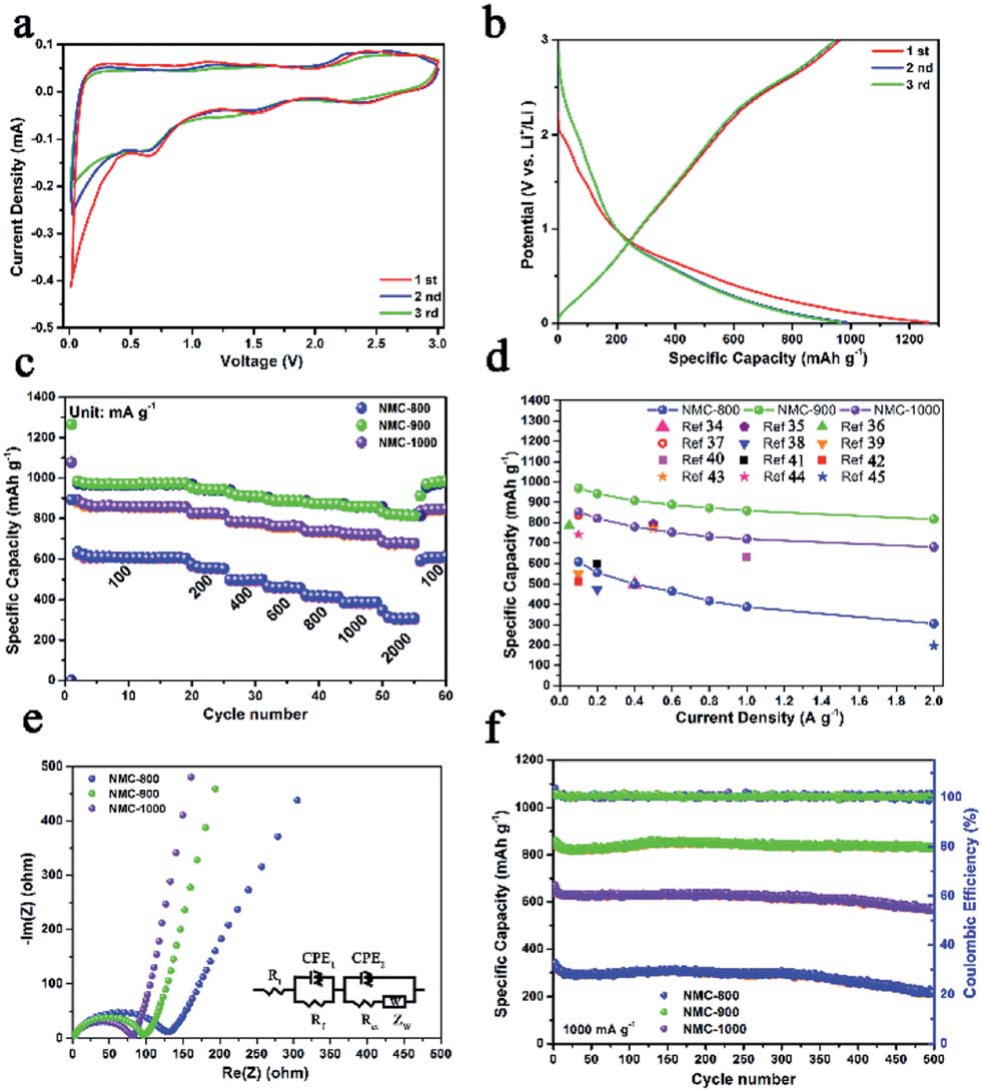
(a) Cyclic voltammetry (CV) curves of NMC-900 at 0.1 mV s^−1^. (b) Charge/discharge processes of NMC-900 in the potential window of 3.0–0.01 V (*vs.* Li^+^/Li) at 0.1 A g^−1^. (c) Rate capabilities and (d) lithium storage capacity of NMC-800, NMC-900, and NMC-1000. (e) Nyquist plots of NMCs. A Randles equivalent circuit displayed in the right side of the figure was used to fit the Nyquist spectra. *R*_i_, *R*_f_, *R*_ct_, *Z*_W_ and CPE denote internal resistance of the test battery, resistance of the SEI layer, charge transfer resistance, Warburg impedance, and constant phase element, respectively. (f) Cycling performance and coulombic efficiency of NMC-800, NMC-900, and NMC-1000.

The lithium storage capacity of NMC-800, NMC-900, and NMC-1000 was investigated using galvanostatic charge–discharge (GCD) and cyclic voltammetry (CV) techniques in half cells with metal Li as the cathode. Fig. 4a shows the typical CV curves of the carbonaceous material. The slope from 2.3 to 3.0 V was related to the effect of heteroatoms on the carbon surface. A large peak at about 1.5 V could be attributed to the extraction of Li^+^ from the defects of carbon (*e.g.* edges and corners from the graphitic layers and heteroatoms), suggesting the presence of numerous defects within carbon.^28,29^ A reduction peak was observed at ∼0.65 V due to the decomposition of the electrolyte on the carbon surface that caused the formation of an SEI layer. The sharp large peak at 0–0.5 V was caused by the intercalation of Li^+^ within carbon, which became weak after the first cycle because of the irreversible intercalation process of Li^+^.^30^ As shown in Fig. 4b and S5,† all the GCD curves of the NMCs displayed a plateau at about 0.9–0.6 V in the initial discharge curve due to the formation of the SEI layer. The first discharge/charge cycle efficiency (CE) of NMC-900 was 77.9% (984 mA h g^−1^/1264 mA h g^−1^), higher than that of NMC-800 (70.1%) and lower than that of NMC-1000 (83.5%). The lowest CE value of NMC-800 could be attributed to the relatively low mesopore proportion with a small and concentrated pore size distribution as well as unsatisfactory electrical conductivity. Furthermore, some special positions in the vicinity of residual H atoms could cause irreversible lithium insertion and/or the reduction of electrolyte, which also influenced the CE value in the first cycle.^31^ Compared with NMC-800, NMC-1000 had a similar specific area, implying that they might have a similar CE. However, its larger average pore size with a higher carbon content and a better electrical conductivity (Table S1†) suggested a more compact structure of the NMC-1000, which might be the reason for the better CE of NMC-1000. Moreover, although NMC-900 exhibited highest electrical conductivity, the middle C and high micropore content limited it to achieve a high cycle efficiency. Then, NMC-900 was further analyzed due to its high electrochemical performance. The first, second, and third discharge capacities of the NMC-900 were 1264, 984, and 975 mA h g^−1^, showing an ultrahigh capacity retention rate of about 77.1%. The corresponding capacity retention rates of NMC-800 and NMC-1000 were 69.4% and 83.6%, respectively. Interestingly, the values of capacity retention were connected with the mesoporous volume and pore size; this implied the superiority of the mesoporous-dominant structure. Moreover, a large mesoporous volume could provide an effective space for Li storage, favorable for promoting the capacity in LIBs.^32^

Because of the good features of porosity, defect structures, and heteroatoms, NMC-900 displayed an excellent Li^+^ storage capacity. The NMC-900 exhibited high specific capacities of 970, 942, 907, 888, 872, 858, and 818 mA h g^−1^ at 0.1, 0.2, 0.4, 0.6, 0.8, 1, and 2 A g^−1^, respectively, showing a favorable rate capability with a capacity retention rate of 88.4% at 2 A g^−1^ (Fig. 4c). This value was much higher than that of the NMC-800 (50.5%) and NMC-1000 (82.4%), which could be attributed to the superior electrical conductivity and developed mesoporous structure.^33^ As shown in Fig. 4d, the average capacities of NMCs at different current densities also visually validated the superior capacity and rate capability of the NMC-900. Compared with the previously reported carbon electrodes,^34–45^ NMC-900 showed a higher capacity than these electrodes for LIBs. Fig. 4e shows the Nyquist plots of NMC-800, NMC-900, and NMC-1000, which have been fitted by an equivalent electric circuit on the right side of Fig. 4e. The semicircle in the middle frequency was the key factor in the Li^+^ transfer efficiency influenced by the CEP_2_ (constant phase element) and *R*_ct_ (charge transfer resistance). The *R*_ct_ value of NMC-800 was 123.6 Ω, much higher than those of NMC-900 (95.4 Ω) and NMC-1000 (78.5 Ω) due to the poorer electronic conductivity and lower mesoporosity of NMC-800. The lowest *R*_ct_ value of NMC-1000 might be due to its super high contents and volume of mesopores with good electronic conductivity. Moreover, all the NMC samples presented good long cycling properties, as shown in Fig. 4f. Even after 500 cycles at 1 A g^−1^, the NMC-900 showed a high Li^+^ storage capacity of about 831 mA h g^−1^, better than 217 mA h g^−1^ for NMC-800 and 566 mA h g^−1^ for NMC-1000. The near-constant coulombic efficiency of approximately 100% for the NMC-900 was caused by high conductivity and abundant mesoporous structure within carbon that provided effective ion diffusion and electron transfer paths.^3^ The corresponding Nyquist plot of the NMC-900 after 500 cycles is shown in Fig. S6.† The *R*_ct_ value of NMC-900 was 129.3 Ω that was close to the original *R*_ct_ value, indicating high cycling stability of NMC-900. The low capacity retention rate for the NMC-1000 electrode might be due to the overgrowth of the HAP nanoparticles that damaged the integrated structure of the NMC-1000. Based on these unique features, NMC-900 was the most promising anode material for application in LIBs.

## Conclusions

A simple, low-priced, and eco-friendly method was explored to prepare nitrogen-doped mesoporous carbons (NMCs) using tortoise shells as a biomass carbon source through HAP-induced self-activation. With a mesoporous-dominated structure, high conductivity, and abundant defects, the as-prepared carbon revealed superior Li^+^ storage performance, high-rate discharge ability, and cyclic stability, which could become a promising anode material in high-rate, stable, and low-cost LIBs.

## Conflicts of interest

There are no conflicts to declare.

## Supplementary Material

RA-008-C8RA02034E-s001
