# Supplementary material for: Mesopore-dominant nitrogen-doped carbon with a large defect degree and high conductivity via inherent hydroxyapatite-induced self-activation for lithium-ion batteries
Source: RSC Adv. 2018 Mar 28;8(22):12204–10. doi: 10.1039/c8ra02034e (PMC9079270; doi:10.1039/c8ra02034e)
Supplement: RA-008-C8RA02034E-s001 [file RA-008-C8RA02034E-s001.pdf]

This journal is © The Royal Society of Chemistry 2018

Mesopore-dominant Nitrogen-doped Carbon with a Large Defects Degree and a High Conductivity *via* Inherent Hydroxyapatite-induced Self-activation for Lithium-ion Battery

Hanwei Wang[1], Chengmin Sheng[1], Tailong Cai[1], Chunde Jin[1], Qingfeng Sun[1], Chao Wang[1*]*

[1]School of Engineering, Zhejiang A&F University, Hangzhou, 311300, China.

[*]Corresponding author: Qingfeng Sun (qfsun@zafu.edu.cn); Chao Wang (chaowangzafu@163.com)

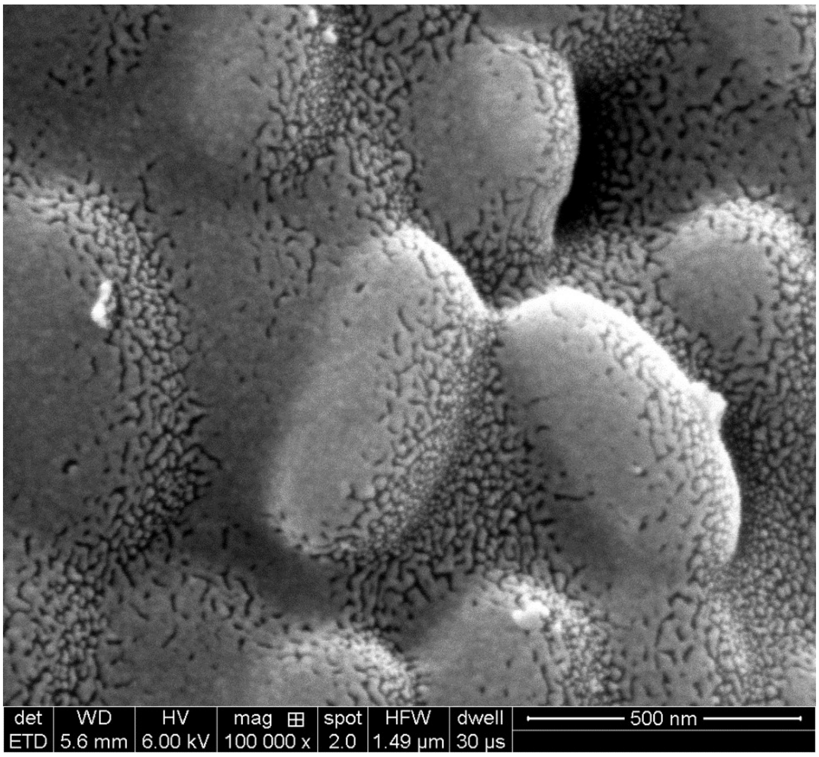

Figure S1. SEM images of NMC-900.

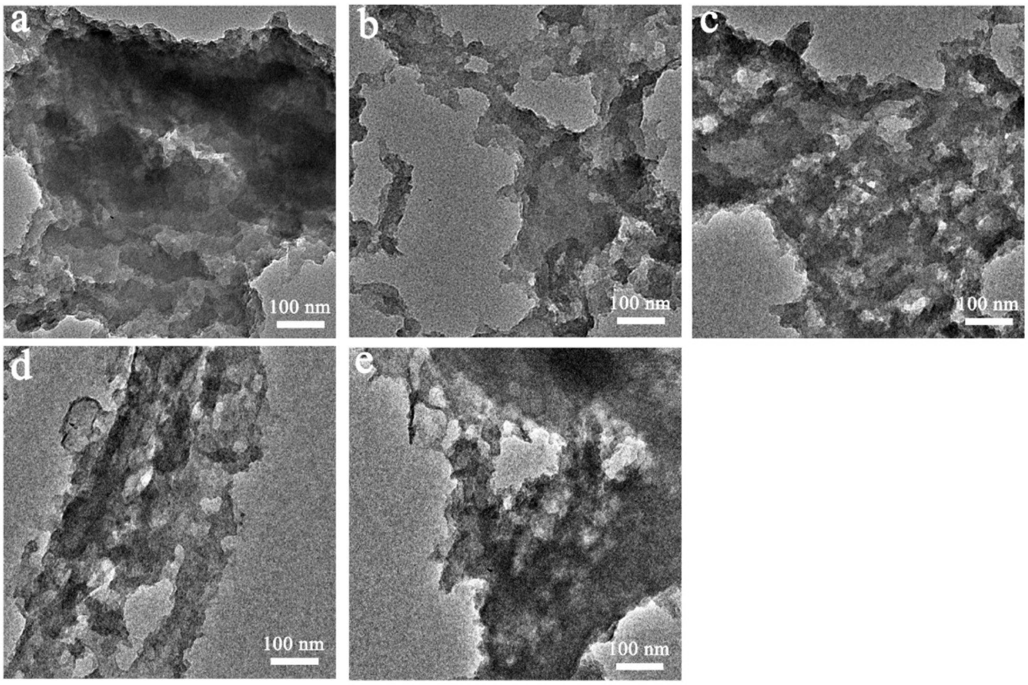

Figure S2. TEM images of (a) NMC-600, (b) NMC-700, (c) NMC-800, (d) NMC-900 and (e) NMC-1000.

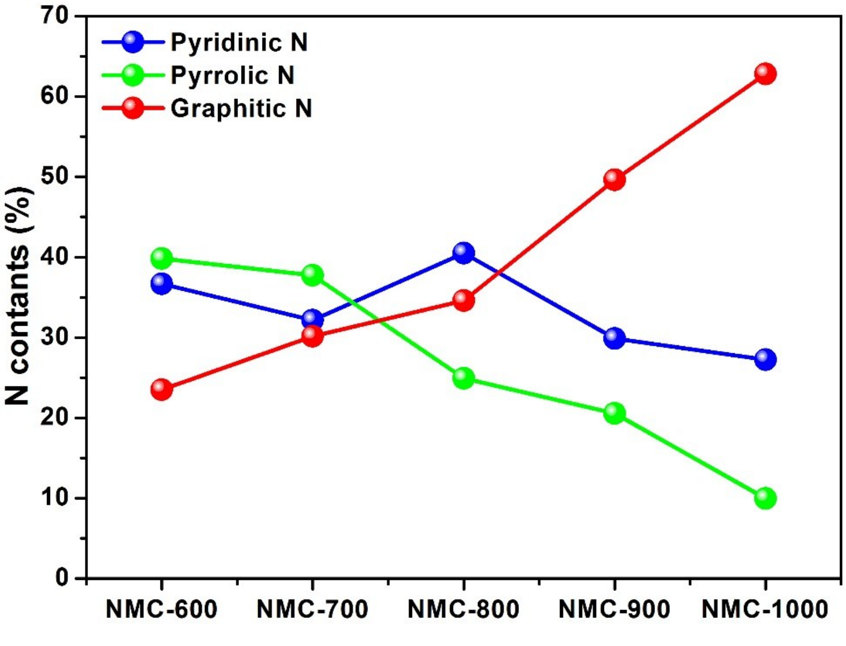

Figure S3. The contents of different N in the NMCs.

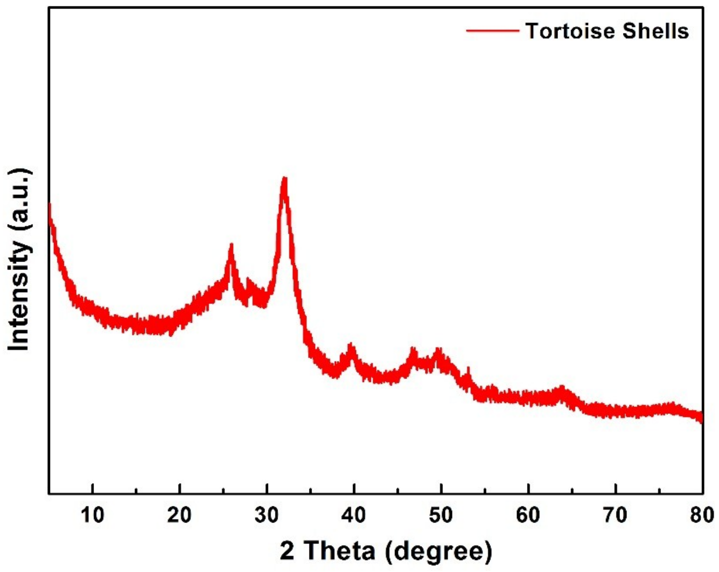

Figure S4. XRD pattern of the tortoise shells.

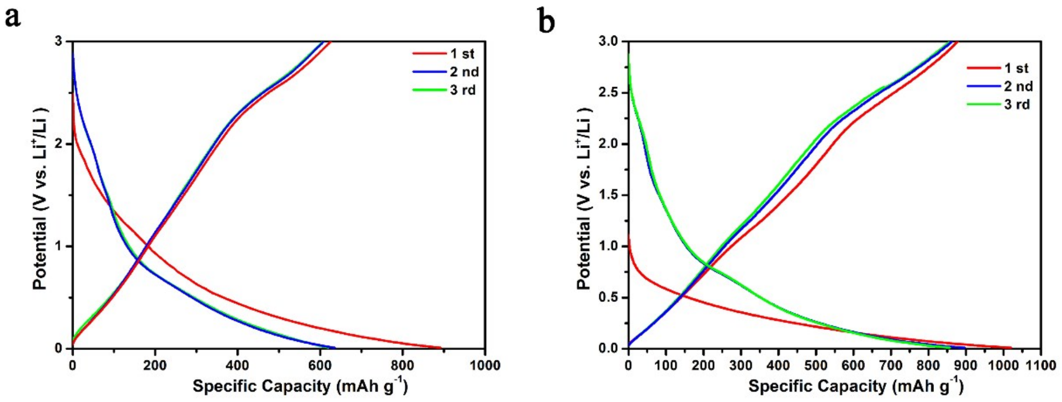

Figure S5. GCD curves of (a) NMC-800 and (b) NMC-1000.

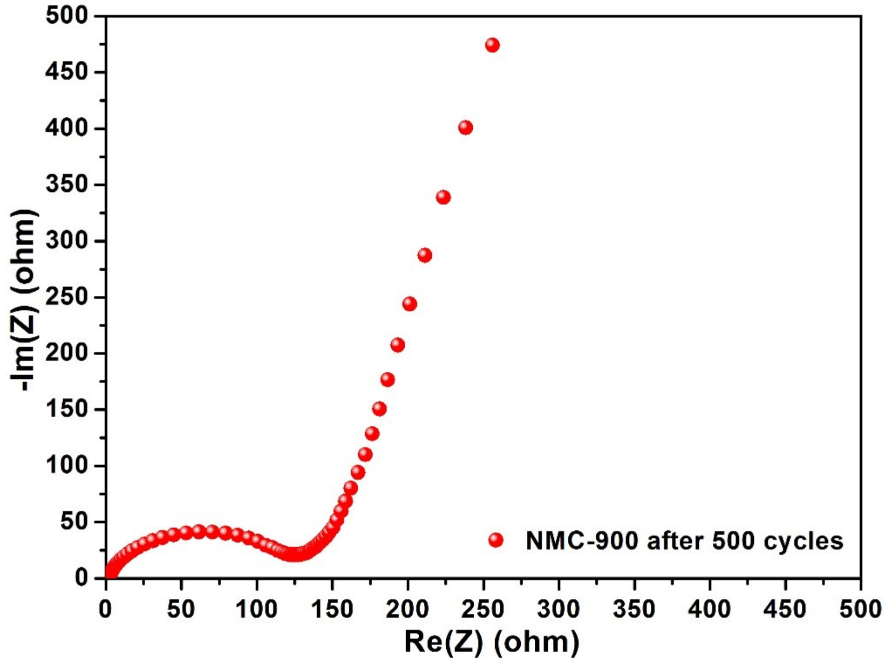

Figure S6. Nyquist plots of NMC-900 after 500 cycles.

Table S1. Elemental analysis of the presented materials.

	XPS (at. %)			Electric conductivity ($S\ m^{-1}$)
	C	N	O	
NMC-600	82.57	9.18	8.25	1.18
NMC-700	84.24	7.02	8.74	107
NMC-800	88.63	6.41	4.96	1047
NMC-900	92.45	3.71	3.84	4382
NMC-1000	93.63	3.16	3.21	2086
